# Spatial variations of women’s home delivery after antenatal care visits at lay Gayint District, Northwest Ethiopia

**DOI:** 10.1186/s12889-019-7050-4

**Published:** 2019-06-03

**Authors:** Araya Mesfin Nigatu, Kassahun Alemu Gelaye, Degefie Tibebe Degefie, Abraham Yeneneh Birhanu

**Affiliations:** 1 Department of Health Informatics, Institute of Public Health, University of Gondar, Gondar, Ethiopia; 2 Department of Epidemiology and Biostatistics, Institute of Public Health, University of Gondar, Gondar, Ethiopia; 3 Climate and Geospatial Research Directorate, Ethiopian Institute of Agricultural Research, Addis Ababa, Ethiopia

**Keywords:** ANC, Cluster, Spatial variation, Home delivery, Northwest Ethiopia

## Abstract

**Background:**

Home delivery is the most frequent childbirth practice in Ethiopia and brings health risks for many mothers and their babies which in turn affecting the whole families. Characterizing the spatial variations and the associated factors of home deliveries after antenatal care visit is necessary to prioritize risks and facilitate geographically based interventions.

**Method:**

A community-based cross-sectional study design was carried out between February and March 2016. A total of 528 women who had just given birth were interviewed face-to-face using a questionnaire. Geo-referenced data were collected using a handheld global positioning system (GPS). The Bernoulli model was applied using the SatScan ™ software to analyze the purely spatial clusters of home deliveries. ArcGIS version 10.1 was used to visualize clusters of home delivery.

**Results:**

The overall proportion of home deliveries was 278(52.7%), and home deliveries had spatial variations. A primary cluster [LLR = 14.54, *p* < 0.001] was detected in village of Safida Giorgis. Secondary clusters were detected in Checheho [LLR = 9.17, *p* < 0.05] and ZurAmba [LLR = 8.51, p < 0.05]. Predictors for home delivery included the distance between the health extension worker’s and mother’s house [AOR = 2.2, 95% CI: 1.1, 4.3], residence [AOR = 3.8, 95% CI: 1.3, 10.9], source of information for ANC [AOR = 0.3, 95% CI: 0.13, 0.7], ANC visits [AOR = 6.1, 95% CI:1.9, 19.3], health education [AOR = 3.4, 95% CI: 1.5, 7.4], decision on place of delivery [AOR = 0.3, 95% CI: 0.1, 0.8], and knowledge on place of delivery [AOR = 0.04, 95% CI: 0.0, 0.1].

**Conclusion:**

The proportion of home delivery after ANC visit was decreasing compared to other studies conducted in the region. In addition, spatial variations of home delivery were observed in the study area. Promoting women’s education and behavioral change communication at the grass root level, provision of the services both at home and health facilities and improving the quality and capacity of the health providers are some of the recommendations forwarded.

## Background

Globally, maternal health is a health agenda of priority. Though the majority of maternal deaths are preventable, every minute of every day, somewhere in the world, women lose their life due to complications related to pregnancy and childbirth [1]. More than half a million women die from causes related to pregnancy and childbirth annually [2]. Home deliveries, which are particularly common in developing countries, bring significant health risks for many mothers and their babies [3].

Of all the health indicators associated with maternal mortality, a remarkable gap exists between affluent and poor women, both between and within countries. Of the globally estimated 289,000 maternal deaths in 2013, a decline of 45% from 1990, the sub-Saharan Africa region alone accounted for 62% (179000) of global deaths followed by Southern Asia at 24% (69000) [2]. Notably, maternal deaths and pregnancy/childbirth-related disablements also severely affect the whole family’s health and wellbeing [4].

Developing regions account for approximately 99% (302000) of the global maternal deaths in 2015, with sub-Saharan Africa alone accounting for roughly 66% (201000), followed by Southern Asia (66000) [1].

In Ethiopia, 84% of births are home deliveries. Decreasing the proportion of home deliveries is an important strategic effort to reduce the maternal mortality rate. Although socio-economic, demographic, and environmental factors influence the prevalence of home deliveries, geographic factors are the major determinants in developing countries [5]. Indeed, the majority of women in Ethiopia live in rural areas where health facilities are inaccessible.

The application of spatial statistics and a geographic information system (GIS) are being progressively employed to identify clusters of health events and to geographically investigate disease patterns [6–8]. Spatial variation analysis is useful to identify the occurrence of disproportion by place and to plan the timely allocation of resources [9]. However, these methods have rarely been employed to analyze the spatial distribution of home deliveries. Considering that characterizing the spatial variation of home delivery is necessary to prioritize risks and facilitate geographically based interventions, we implemented these methods in the current study.

## Methods

### Study design and settings

**Fig. 1:**
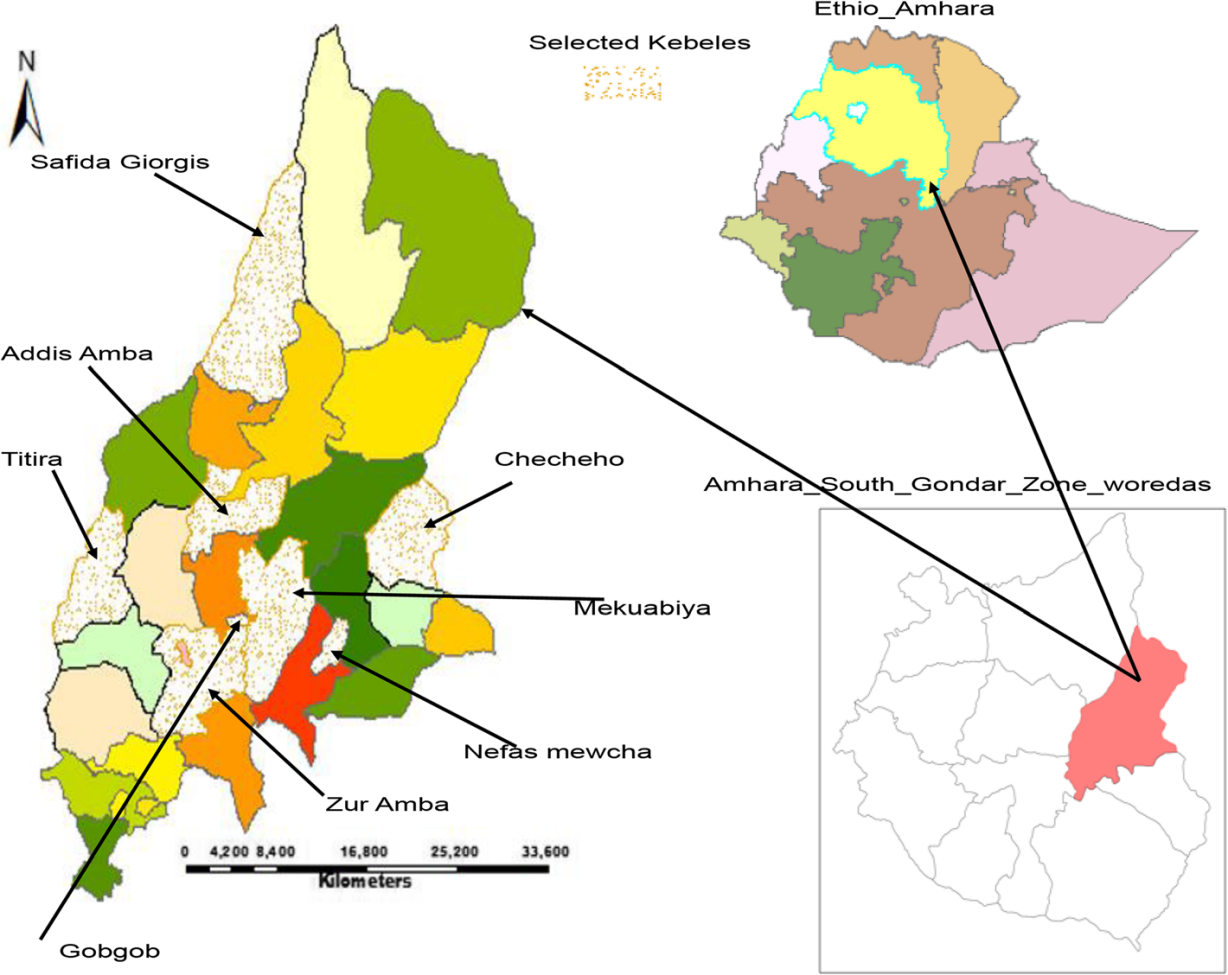
Map of study area Lay Gayint district, Northwest Ethiopia; 2016

This was a community-based cross-sectional study that assessed the spatial variation and associated factors of women’s home deliveries after ANC visits in Lay Gayint district, Amhara Regional State, northwest Ethiopia. The study was conducted between February 15, 2016, and March 13, 2016. The district has 29 kebeles (the smallest local administrative units). Of these, 25 are rural kebeles. Healthcare services in the district are provided to the community by 9 health centers, 38 health posts, and 1 primary hospital (Fig. 1). Of the estimated population of 230,561, 116,322(50.4%) are women and 31,511(13.7%) are urban inhabitants. The district has an area of 1522.43 km^2^ with a population density of 151.44 persons per square kilometer. Of the 116,322 females, 54,956 (47.2%) were within the reproductive age at the time of the study. The estimated annual delivery number within this period was 1852 births [10].

### Sample size and sampling procedures

The sample size was calculated using a single population proportion formula with an assumption of a 95% confidence interval, 5% margin of error, 1.5 design effect, 10% non-response rate, and 68.4% home delivery prevalence [11]. The final sample size was 548.

A total of 1048 estimated the number of deliveries was identified from all kebeles. Using proportional allocation followed by a simple random sampling technique, 548 women who gave birth during the previous 12 months prior to the study period and had one or more ANC visits were involved from six rural and two urban kebeles (25% from the total 29 kebeles). A document review was carried out at ANC clinics to identify eligible participants, and the logbook of health extension workers was crosschecked to verify eligible participants. The sample size was allocated proportionally to each ANC clinic according to the total number of deliveries in the study period. Eligible participants were reached in person using their addresses, obtained from the ANC clinics and health extension worker’s logbook.

### Operational definition

Home delivery was defined as unattended childbirth in a non-clinical setting that takes place in a residence rather than a health facility.

Skilled professionals are health personnel who is able to manage normal and complicated labor. The distance was defined as the distance between the respondents’ home to the nearest health facility and health extension worker’s home. It was calculated by using an arc GIS nearby function.

### Data collection procedures

We implemented a structured and pre-tested interviewer-administered questionnaire that comprised items concerning socio-demographic, accessibility, behavioral and obstetric factors. The questionnaire was prepared in English and then translated to Amharic (the local and national language) and then translated back to English by language experts to check its consistency. The location data were taken at women’s homes, the health extension worker’s home and at health facilities using a handheld GPS with a precision of 3 m. Shapefiles were obtained from the Central Statistical Agency (CSA) [12]. The location data and attributes were cross-linked with kebeles using the Arc GIS version 10.1 software [13] and characterized by unique latitude and longitude coordinates.

### Data quality control measures

The training was given for data collectors and supervisors on the objectives of the study, data collection, and keeping the confidentiality of respondents prior to data collection. The completeness of the questionnaire was checked.

### Statistical analysis

Data were entered, edited, and cleaned using EpiInfo version 7 and exported to SPSS version 20 for further statistical analysis. The binary logistic regression method was used to analyze the association between variables including distance from health facility, distance from the nearest HEW’s home, residence, age, education, source of information about ANC, benefit of ANC, ANC visits, health education, decision on place of delivery, knowledge on place of delivery, gravidity, parity, wealth index, and place of delivery. Variables found to have an association with the dependent variable at a *p*-value of less than 0.2 in the bivariable analysis were entered into the multivariable binary logistic regression model for controlling the possible effects of confounders. Variables that had a significant association were identified on the bases of the odds ratio (OR), 95% CI, and a p-value of less than 0.05.

### Statistical methods for spatial analysis

The Bernoulli model was applied using SaTScan™ software to analyze the spatial distribution of home deliveries following an ANC visit [14]. The spatial scan statistic used a circular scan window to scan the population at risk by moving across the study area. In this study, the size of the scan window was set to < 10% of the population at risk to scan for small clusters. The likelihood function was maximized over all windows, and the window with the maximum likelihood constituted the most likely cluster [15].

The non-overlapping possibility was used to identify secondary clusters. Clusters with significant large likelihood ratios were recognized. The *p*-value was generated using Monte Carlo replications. The number of Monte Carlo replications was set to 999 to ensure acceptable power for defining clusters. A p-value of less than 0.05 was considered to be statistically significant. The specific locations of clusters were calculated in terms of relative risks (RRs). A cluster with RR > 1 indicates an increased risk compared to the risk outside that cluster [14, 15].

Study participant’s location data were linked to a cluster polygon map of the kebeles using Arc GIS software. The near function method was used to calculate the distance of each study participant’s home from the nearest health extension worker’s home and the nearest health facility.

### Local Moran’s I

Anselin’s Local Moran’s I statistic was used to identify clusters of features with values similar in magnitude, to identify outliers by comparison to neighboring features, and to map the residuals after building the regression model and to evaluate whether or not spatial pattern existed in the residuals.

### Ethical considerations

Ethical clearance was obtained from the Institutional Review Board of the University of Gondar. A permission letter was also obtained from the Amhara National Regional State Health Bureau. Informed consent was held to each study participant about the benefits and risks of the study. Participant records were coded on each respective questionnaire and, to maintain confidentiality, were only accessible to the research team members.

## Results

### Socio-demographic characteristics

A total of 528 mothers who had at least one ANC visit for their last pregnancy and had given birth within the last 12 months were interviewed, and we received a response rate of 96.4%. Of the total number of participants, 385 (73%) were from rural areas; 392 (74.2%) were 20 to 34 years of age, 484 (91.7%) were married, and 511 (96.8%) were Orthodox Christians. The mean age of participants was 28 ± 6 years. Of these, 460 (87.1%) were housewives, 243 (46%) were unable to read and write, and 246 (46.6%) were annual income below the national average (Table 1).

**Table 1:** Socio-demographic characteristics of women in the Lay Gayint district, Northwest Ethiopia; 2016

Variables	Residence		Total (*n* = 528) No (%)
	Urban (*n* = 143)	Rural (*n* = 385)	
Age (in years)			
< 20	4 (2.8)	18 (4.7)	22 (4.2)
20–34	116 (81.1)	276 (71.7)	392 (74.2)
35–49	23 (16.1)	91 (23.6)	114 (21.6)
Marital status			
Married	124 (86.7)	360 (93.5)	484 (91.7)
Single	12 (8.4)	12 (3.1)	24 (4.6)
Other^a^	7 (4.9)	13 (3.4)	20 (3.8)
Occupation			
Housewife	93 (65.0)	367 (95.3)	460 (87.1)
Merchant	18 (12.6)	1 (0.3)	19 (3.6)
Civil servant	15 (10.5)	3 (0.8)	18 (3.4)
Daily laborer	8 (5.6)	6 (1.5)	14 (2.7)
Student	7 (4.9)	6 (1.5)	13 (2.5)
Farmer	1 (0.7)	1 (0.3)	2 (0.4)
Other^b^	1 (0.7)	1 (0.3)	2 (0.4)
Education			
Unable to read and write	24 (16.8)	219 (56.9)	243 (46.0)
Able to read and write	8 (5.6)	32 (8.3)	40 (7.6)
Primary school (1–8)	39 (27.3)	105 (27.3)	144 (27.3)
Secondary school (9–12)	50 (34.9)	27 (7.0)	77 (14.6)
College and above	22 (15.4)	2 (0.5)	24 (4.50
Religion			
Orthodox	130 (91)	381 (98.9)	511 (96.8)
Muslim	10 (6.9)	4 (1.0)	14 (2.6)
Protestant	3 (2.0)	0	3 (0.6)
Wealth Index			
Poor	46 (26.1)	130 (73.9)	176 (33.3)
Medium	32 (30.2)	74 (69.8)	106 (20.1)
Rich	65 (26.4)	181 (73.6)	246 (46.6)

other^a^ = Separated, widowed, divorced

other^b^ = retired, unemployed

### Place of delivery and reasons for home delivery

**Fig. 2:**
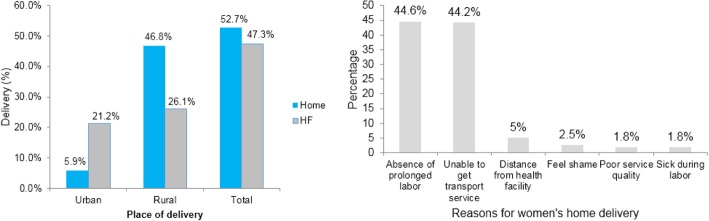
Place of delivery and reasons for home delivery in Lay Gayint district, Northwest Ethiopia; 2016

**Fig. 3:**
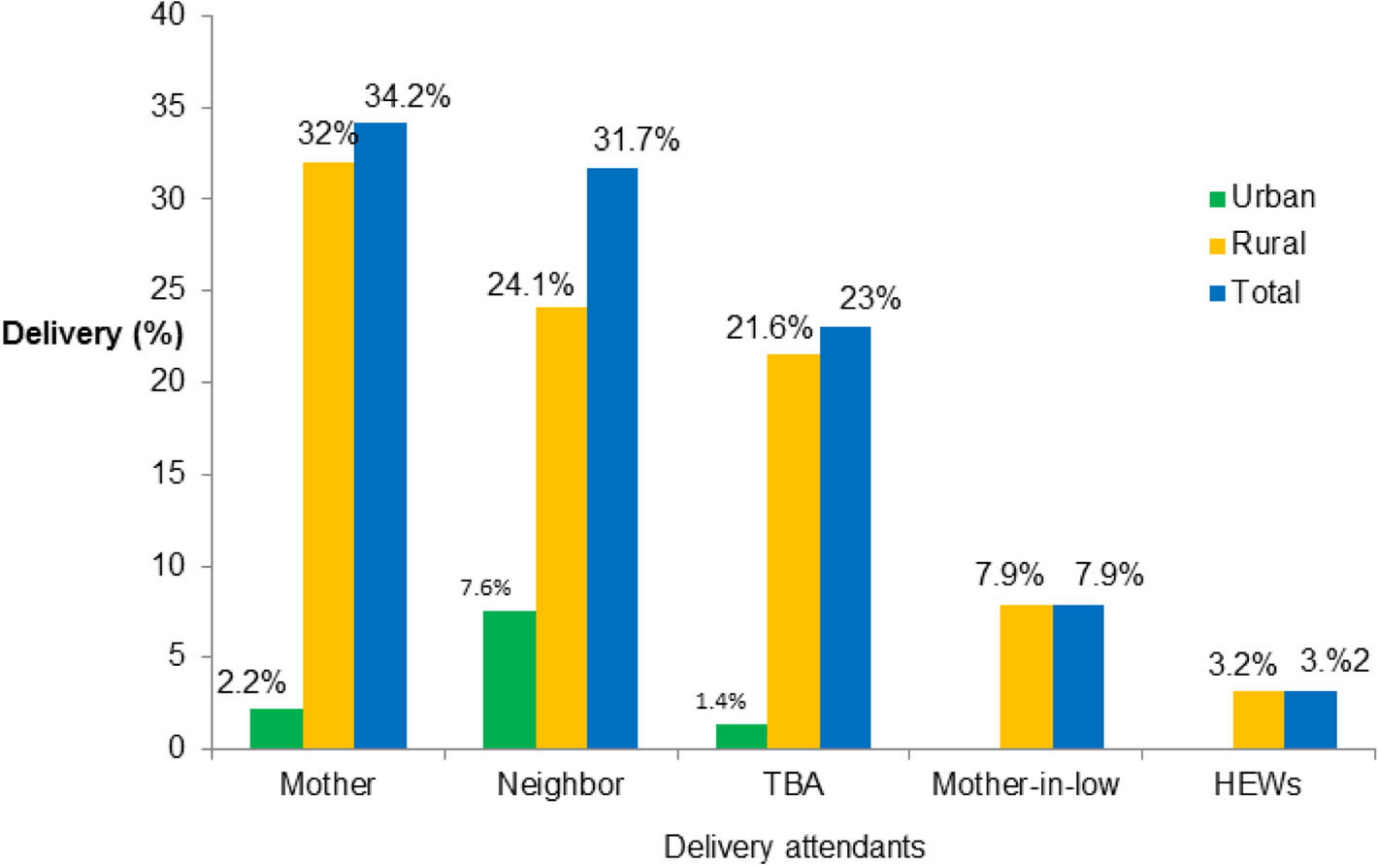
Home delivery attendants by residence in Lay Gayint district, Northwest Ethiopia; 2016

Of the 528 mothers, 278 (52.7%) gave birth at their home (95% CI: 48.5, 56.6%). The most common reasons for home delivery were inaccessible transport services (*n* = 123, 44.2%), short duration of labor (*n* = 124, 44.6%), shame (*n* = 7, 2.5%), and distance from their home to health facility (*n* = 14, 5%) (Fig. 2). Of the women who had home delivery, 95 (34.2%) were attended to by their mothers, 88 (31.7%) by a neighbor, and 64 (23%) by traditional birth attendants (Fig. 3).

### Obstetrics characteristics

**Fig. 4:**
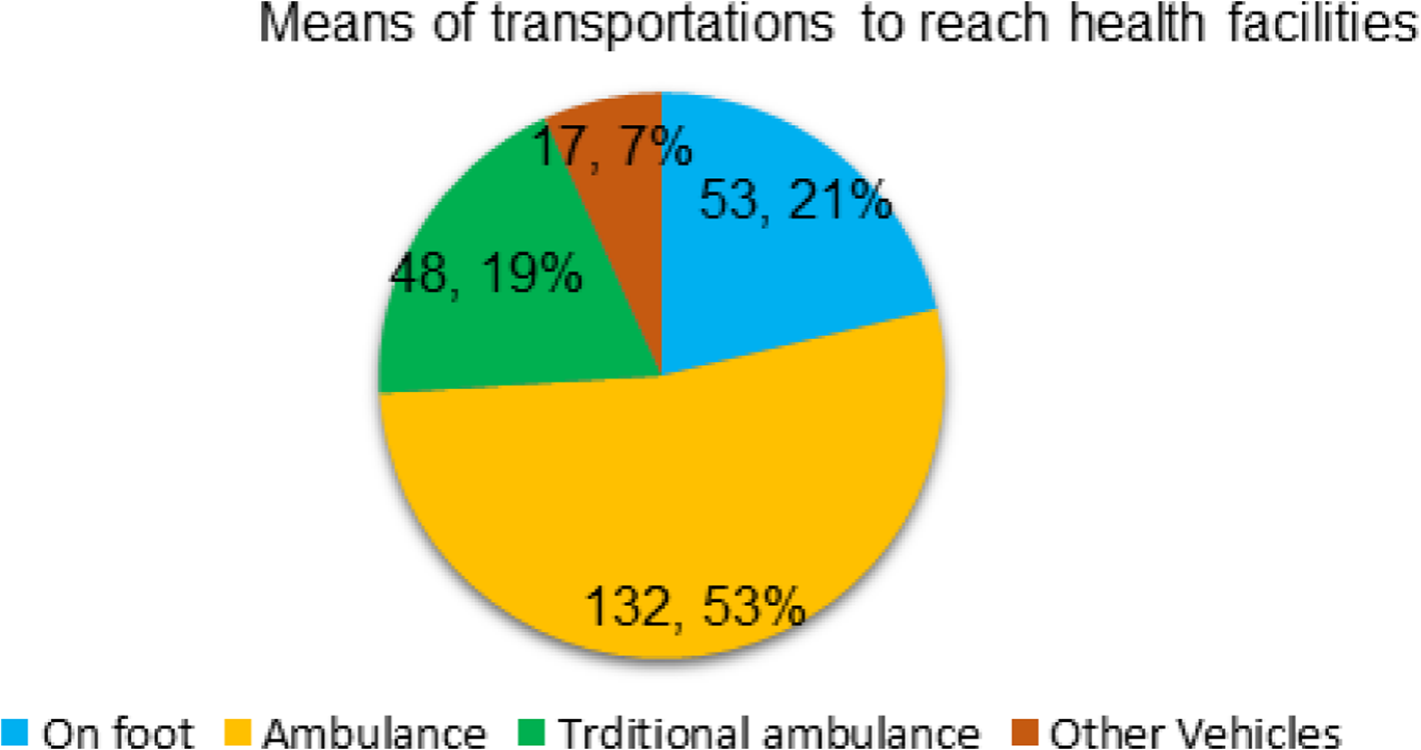
Means of transportations to reach health facilities in Lay Gayint district, Northwest Ethiopia; 2016

Of the women who came from urban areas (*n* = 143), 72 (50.3%) visited ANC services four times; 316 (59.9%) had two to four pregnancies, and 320 (60.6%) had two to four parities. Of the total home deliveries (*n* = 278), 247 (88.8%) were from rural areas. For women, who had home births, the mean distance to the nearest health facility was 2.46 ± 1.95 km, and the mean distance to a health extension worker’s home was 1.93 ± 1.48 km. Of women from urban areas, 143 (100%) resided within 2.5 km to the nearest health facility and 132 (92.3%) within 2.5 km to a health extension worker’s home. From rural areas respondents, 151 (39.2%) and 237 (61.6%) resided within 2.5 km to the nearest health facility and health extension worker’s home respectively (Table 2). Of those who gave birth at a health facility, 132 (53%) had accessed an ambulance service (Fig. 4).

**Table 2:** Obstetric and location characteristics of women in the Lay Gayint district, Northwest Ethiopia; 2016

Variables	Residence		Total No (%)
	Urban (*n* = 143)	Rural (*n* = 385)	
ANC visits			
1	7 (4.9)	67 (17.4)	74 (14.0)
2–3	64 (44.8)	178 (46.2)	242 (45.8)
> 4	72 (50.3)	140 (36.4)	212 (40.2)
Gravidity			
1	39 (27.3)	72 (18.7)	111 (21)
2–4	92 (64.3)	225 (58.4)	316 (59.9)
> 5	12 (8.4)	88 (22.9)	101 (19.1)
Parity			
1	39 (27.3)	72 (18.7)	111 (21)
2–4	92 (64.3)	228 (59.2)	320 (60.6)
> 5	12 (8.4)	88 (22.9)	97 (18.4)
Distance from the nearest HF			
< =2.5 km	143 (100)	151 (39.2)	294 (55.7)
2.51–5 km	0	197 (51.2)	197 (37.3)
> 5 km	0	37 (9.6)	37 (7)
Distance from the nearest HEW residence			
< =2.5 km	132 (92.3)	237 (61.6)	369 (69.9)
2.51–5 km	11 (7.7)	132 (34.3)	143 (27.1)
> 5 km	0	16 (4.1)	16 (3.0)

*HF* Health facility, *HEW* Health Extension Worker

### Information and behavioral characteristics of respondents

The source of information for ANC services were: health care professionals 215 (40.7%), health extension workers 162 (30.7%), radio/Television 74 (14%), relatives and friends 77 (14.6%). About 284 (53.8%) of them considered this the information to be helpful to both mothers and their children. Four hundred and thirteen (78.2%) women decided their place of delivery jointly with their husbands. Only, 106 (20%) women received health education during their ANC visits (Table 3).

**Table 3:** Behavioral characteristics of women and their source of information in the Lay Gayint district, Northwest Ethiopia; 2016

Variables	Frequency	Percent
Source of information about ANC		
Health institution by health care workers	215	40.7
Health extension workers	162	30.7
Radio/Television	74	14.0
Relatives and friends	77	14.6
Received health education during ANC visit		
Yes	106	20.0
No	191	36.2
Do not remember	231	43.8
Benefit of ANC		
Both for mother’s and child’s health	284	53.8
For a child’s health	184	34.8
For mother’s health	60	11.4
The decision on the place of delivery		
Both wife and husband	413	78.2
Wife only	82	15.5
Husband only	33	6.3
Is there a difference of giving birth at health facility instead of home?		
Yes	340	64.4
No	12	2.3
I don’t know	176	33.3

### Spatial variation of home deliveries

**Fig. 5:**
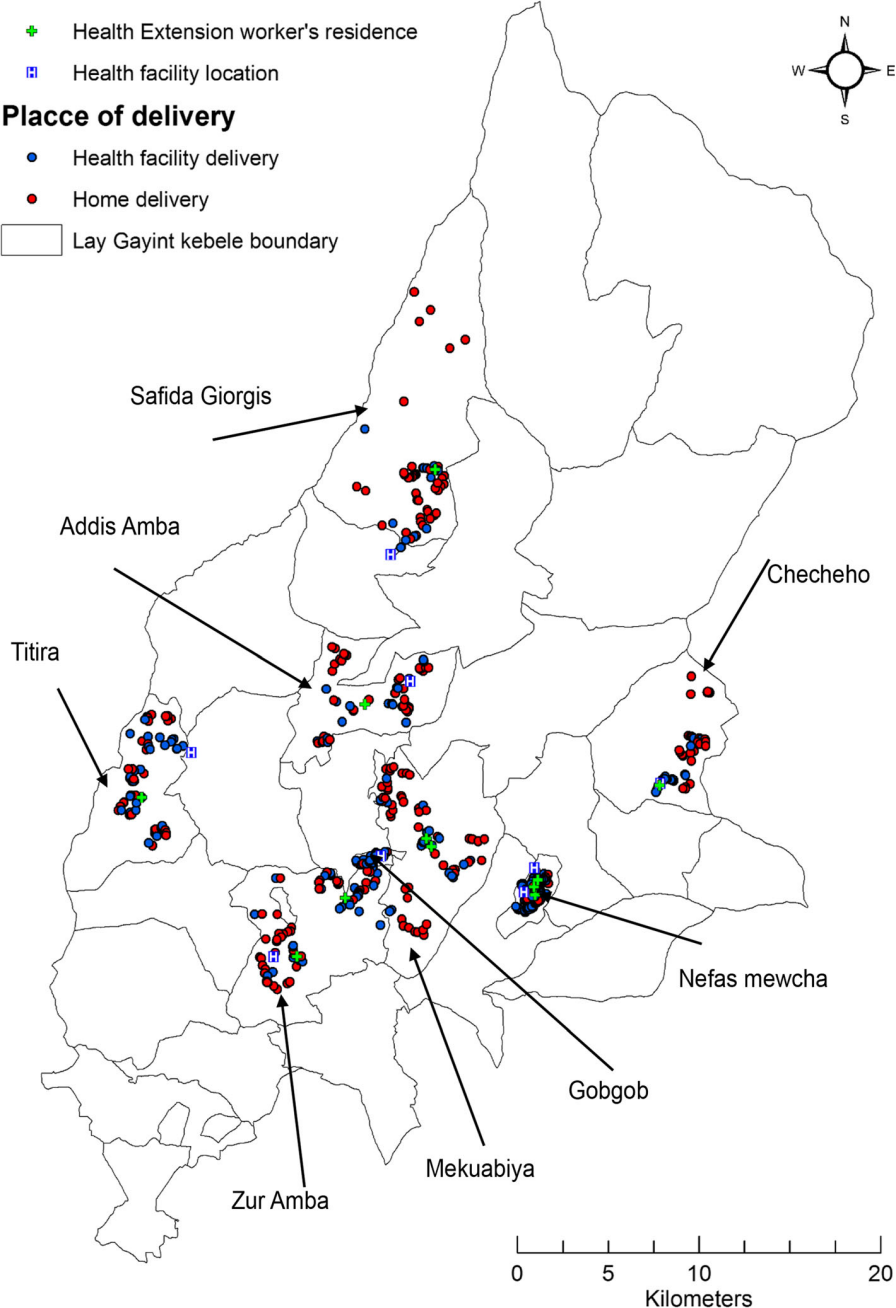
Women’s residence, health facilities and health extension worker’s location in Lay Gayint district, Northwest Ethiopia; 2016

**Fig. 6:**
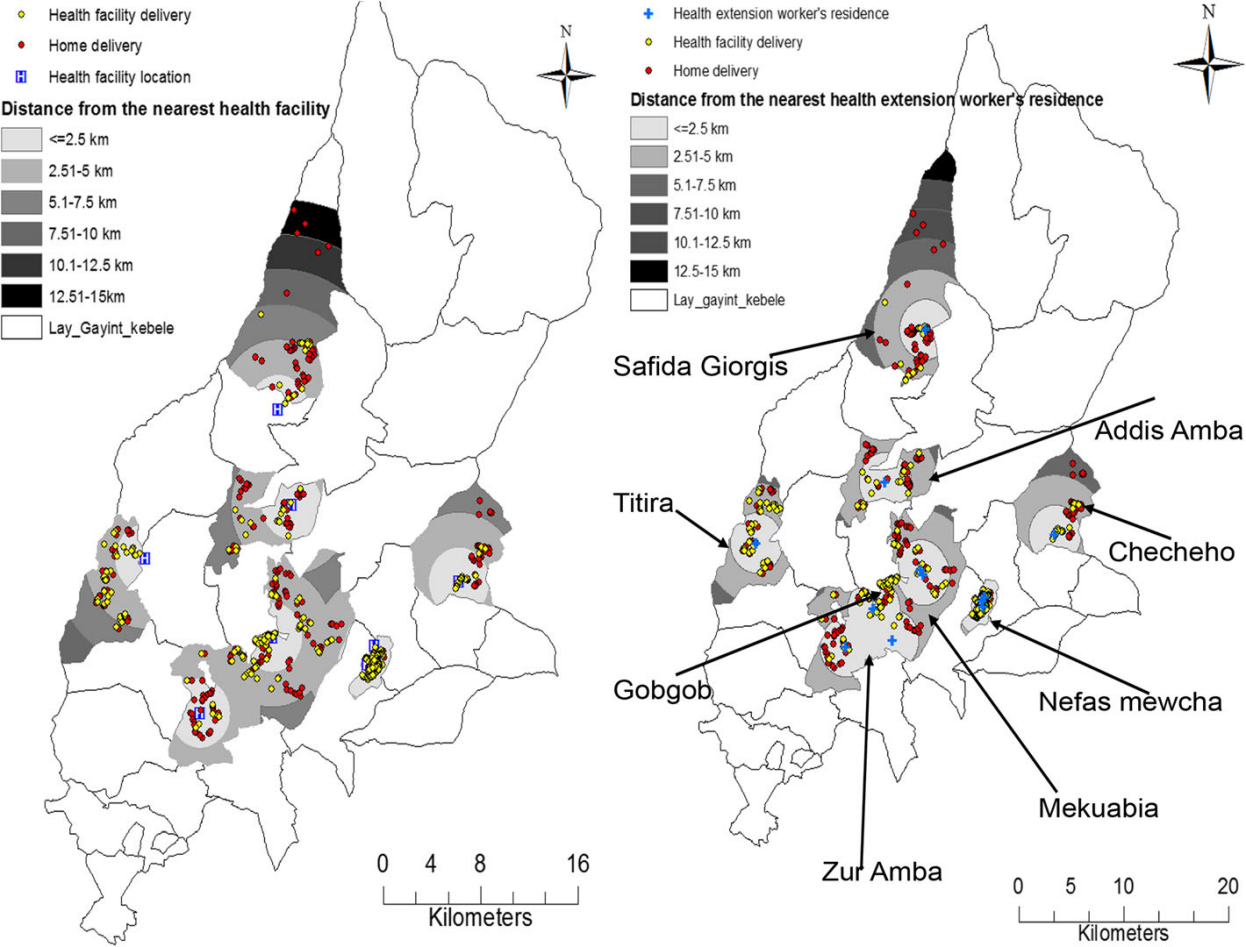
Buffer analysis map for women’s place of delivery in Lay Gayint district, Northwest Ethiopia; 2016

**Fig. 7:**
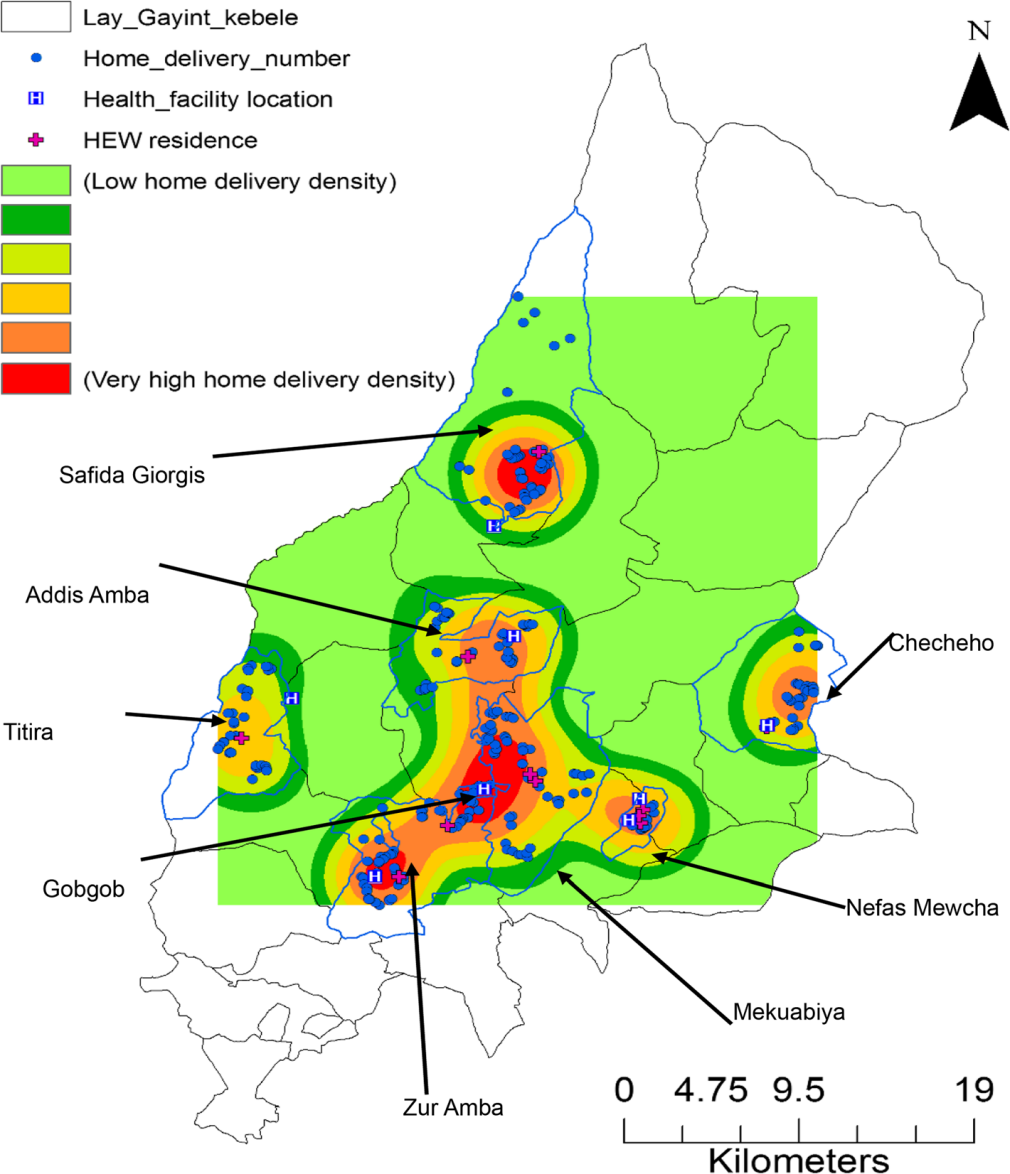
Kernel density analysis map for women’ place of delivery in Lay Gayint district, Northwest Ethiopia; 2016

Spatial cluster analysis revealed that home deliveries have shown spatial variation (Fig. 5). Buffer analysis demonstrated that the majority of home deliveries were within 5 km of the nearest health facility and health extensions worker’s home (Fig. 6). We also demonstrated hot spot areas of home deliveries and variations across study areas (Fig. 7).

### Spatial scan statistics

**Fig. 8:**
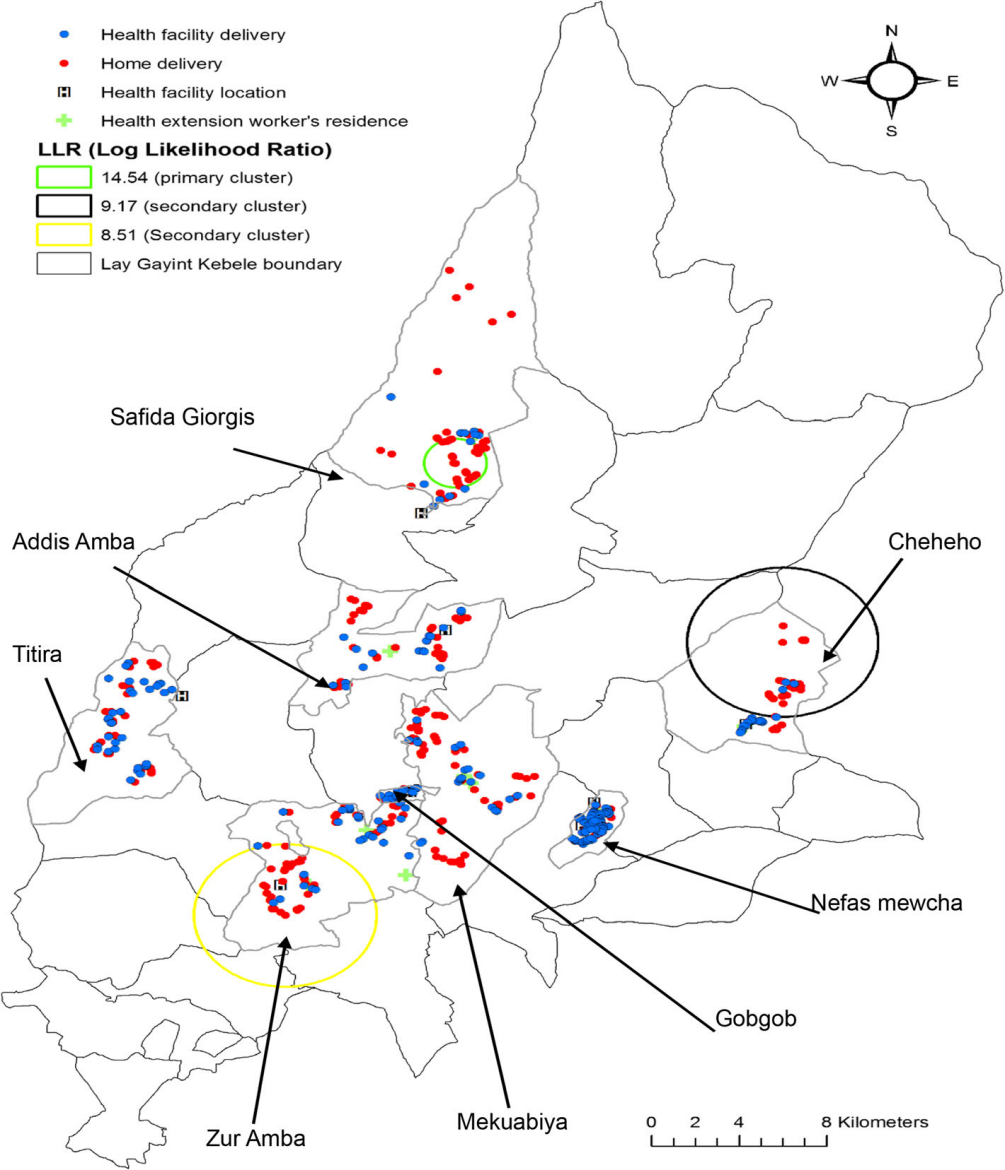
Cluster detection analysis of home delivery in Lay Gayint district, Northwest Ethiopia; 2016

The highest proportion of home deliveries was in the Mekuabiya and ZurAmba kebeles (*n* = 50, 18%). Ten spatial clusters of home deliveries were identified. The most likely spatial clusters were detected (LLR = 14.5, *p* < 0.001) in the Safida Giorgis kebele in which there were 22 (4%) births (Fig. 8). Secondary clusters were mainly located in the Checheho [LLR = 9.2, *p* < 0.05] and ZurAmba [LLR = 8.5, *p* < 0.05] kebeles (Table 4).

**Table 4:** Significant clusters for home delivery in the Lay Gayint district, Northwest Ethiopia; 2016

Type of cluster	Total # of population	Total # of cases	RR	Cases(%)	LLR	Radius (km)
Most likely cluster*	22	22	2	100	14.5	1.4
Secondary cluster*	28	25	1.8	89.3	9.2	4.4
Secondary cluster*	37	31	1.7	83.8	8.5	4.2

*Primary cluster at *p* < 0.001; *Secondary cluster at *p* < 0.05

*LLR* log likelihood ratio, *RR* relative risks

### Local Moran’s I results

**Fig. 9:**
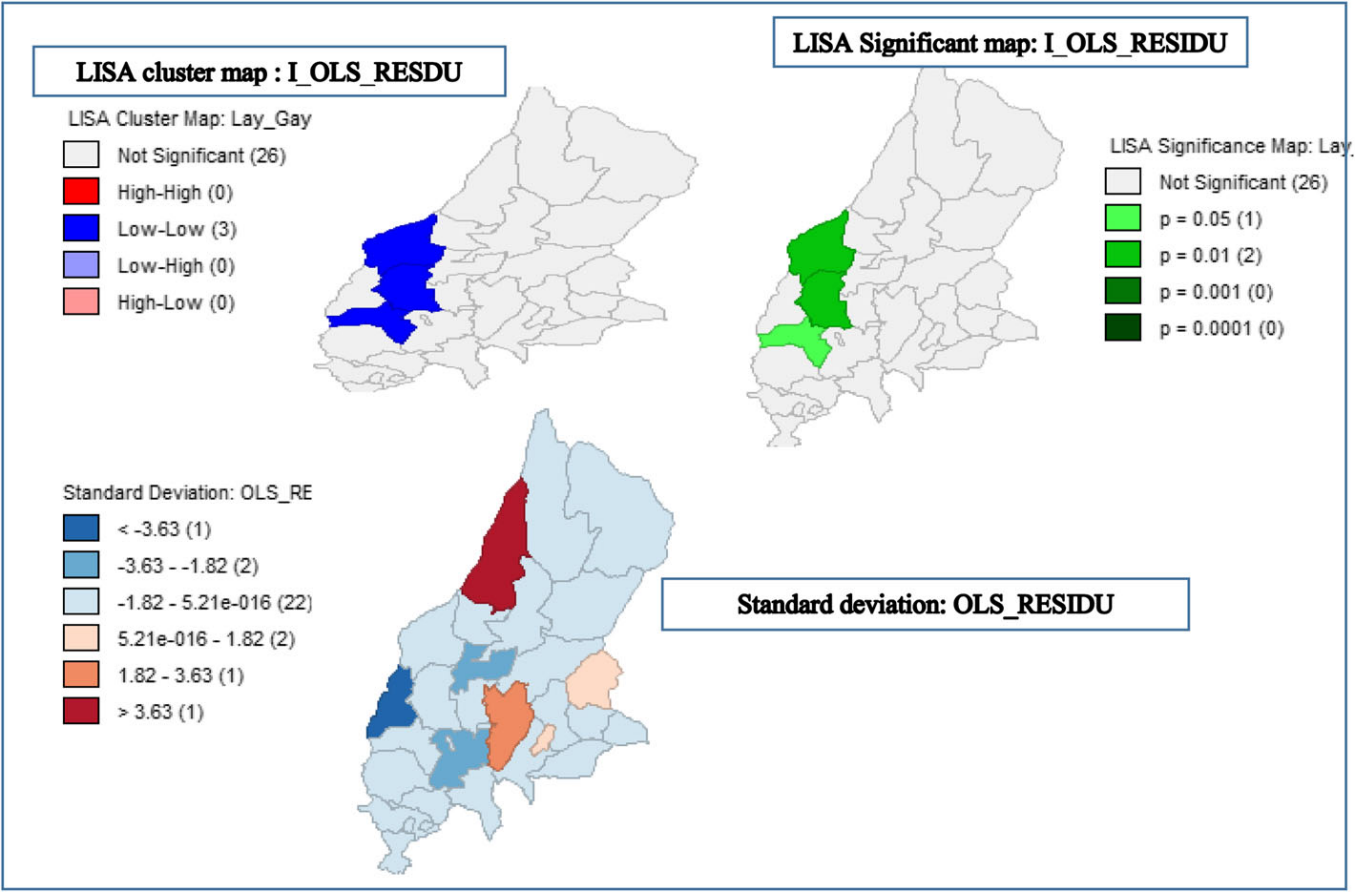
Local Moran’s I for women’s home delivery in Lay Gayint district, Northwest Ethiopia; 2016

Figure 9 shows that the Local Moran’s I result agree with areas with clustering of home deliveries. However, there are kebele that the Local Moran’s I classified as not significant or low/dissimilar. One cluster classified as insignificant by the Local Moran’s I technique was shown by SaTScan. On interpreting this map, high values (large positive residuals) indicate model under prediction. At the very least, these clusters and spatial outliers are places of interest that merit further studies (Fig. 9).

### Factors associated with home deliveries

Women who lived within 2.5–5 km of a health extension worker’s home (AOR = 2.2, 95% CI: 1.1, 4.3) were twice more likely to deliver at home than those who resided within 2.5 km. Women from rural areas (AOR = 3.8, 95% CI: 1.3, 10.9) were four times more likely to give birth at home compared to women from urban areas.

Mothers who had one ANC visit (AOR = 6.1, 95% CI: 1.9, 19.3) were six times more likely to deliver at home compared to women who had four or more ANC visits. Women who did not receive health education (AOR = 3.4, 95% CI: 1.5, 7.4) were three times more likely to deliver at home compared to those who obtained health education during ANC visits.

Mothers who had received a place of delivery information from health facilities (AOR = 0.3, 95% CI: 0.1, 0.7) were 70% less likely to deliver at home compared to their counterparts. Mothers who decided the place of delivery jointly with her husband (AOR = 0.3, 95% CI: 0.1, 0.8) were 70% less likely to give birth at home compared to mothers who decided alone. Women who had knowledge about the place of delivery (AOR = 0.04, 95% CI: 0.02, 0.09) were 96% less likely to give birth at home compared to their counterparts (Table 5).

**Table 5:** Bivariable and multivariable analysis of home delivery in the Lay Gayint, Northwest Ethiopia; 2016

Variables	Place of delivery		COR (95% CI)	AOR (95% CI)
	Home	HF		
Distance from HF location				
< =2.5 km	124 (42.2)	170 (57.8)	1.0	1.0
2.51–5 km	128 (65)	69 (35)	2.5 (1.8, 3.7)*	1.1 (0.6, 2.1)
> =5 km	26 (70.3)	11 (29.7)	3.2 (1.5, 6.8)*	1.2 (0.3, 3.9)
Distance from the nearest HEW home				
< =2.5 km	168 (45.8)	199 (54.2)	1.0	1.0
2.51–5 km	97 (67.8)	46 (32.2)	2.5 (1.6, 3.7)*	2.2 (1.2, 4.3)**
> =5 km	13 (72.2)	5 (27.8)	3.1 (1.1, 8.8)*	1.4 (0.3, 7.0)
Place of residence				
Urban	29 (20.3)	114 (79.7)	1.0	1.0
Rural	249 (64.7)	136 (35.3)	7.2 (4.6, 11.4)*	3.8 (1.3, 10.9)***
Age				
< 20	10 (45.5)	12 (54.5)	1.0	1.0
20–34	199 (50.8)	193 (49.2)	1.2 (0.5, 2.9)	1.1 (0.3, 4.6)
> =35	69 (60.5)	45 (39.5)	1.8 (0.7, 4.6)	0.8 (0.2, 3.8)
Level of education				
Unable to read and write	159 (64.4)	88 (35.6)	12.7 (3.4, 43.6)*	0.3 (0.1, 1.7)
Able to read and write	21 (52.5)	19 (47.5)	7.7 (1.9, 30.1)*	0.2 (0.0, 1.6)
Primary (1–8)	76 (54.3)	64 (45.7)	8.3 (2.4, 29.1)*	0.7 (0.1, 3.7)
Secondary (9–12)	19 (24.7)	58 (75.3)	2.3 (0.6, 8.5)	0.7 (0.1, 3.7)
College and above	3 (12.5)	21 (87.5)	1.0	1.0
Source of information for ANC				
Health institution	119 (55.3)	96 (44.7)	0.5 (0.3, 0.8)*	0.3 (0.1, 0.8)**
HEW	82 (50.6)	80 (49.4)	0.4 (0.2, 0.7)*	0.4 (0.2, 1.1)
Radio/TV	21 (28.4)	53 (71.6)	0.2 (0.1, 0.3)*	0.4 (0.1, 1.2)
Friends and relatives	56 (72.7)	21 (27.3)	1.00	1.00
Benefit of ANC				
Maternal health	50 (83.3)	10 (16.7)	1.00	1.00
Child health	134 (72.8)	50 (27.2)	0.5 (0.3, 1.1)	1.1 (0.4, 2.9)
Both mother and child	94 (33.1)	190 (66.9)	0.1 (0.0, 0.2)*	0.4 (0.1, 1.0)
ANC visits				
1 visit	70 (88.6)	9 (11.4)	18.6 (8.7, 39.6)*	6.2 (1.9, 19.3)***
2–3 visits	147 (60.7)	95 (39.3)	3.7 (2.5, 5.5)*	2.0 (1.1, 3.4)**
≥ 4visits	61 (29.5)	146 (70.5)	1.0	1.0
Health education				
Yes	34 (32.1)	72 (67.9)	1.0	1.0
No	131 (68.6)	60 (31.4)	4.6 (2.7, 7.7)*	3.5 (1.6, 7.7)***
I don’t know	113 (48.9)	118 (51.1)	1.0 (1.252, 3.286)*	2.7 (1.3, 5.7)**
A decision on the place of delivery				
Myself	68 (82.9)	14 (17.1)	1.0	1.0
My husband	18 (54.5)	15 (45.5)	0.3 (0.1, 0.6)	0.3 (0.1, 1.2)
Both of us	192 (46.5)	221 (53.5)	0.2 (0.1, 0.3)*	0.3 (0.1, 0.9)**
Knowledge on place of delivery				
Yes	104 (30.6)	236 (69.4)	0.04 (0, 0.1)*	0.04 (0.0, 0.1)***
No	174 (92.6)	14 (7.4)	1.0	1.0
Gravidity				
1	41 (36.9)	70 (63.1)	1.0	1.0
2–4	176 (55.6)	140 (44.3)	2.2 (1.4, 3.3)*	0.4 (0.1, 1.1)
> 5	61 (60.4)	40 (39.6)	261 (1.5, 4.5)*	0.4 (0.0, 6.5)
Parity				
1	41 (50)	41 (50)	1.0	1.0
2–4	179 (55.9)	141 (44.1)	2.2 (1.4, 3.4)*	2.2 (0.1, 35.7)
> 5	58 (59.8)	39 (40.2)	2.5 (1.5, 4.4)*	1.9 (0.9, 8.3)
Wealth Index				
Poor	50 (27)	135 (73)	0.2 (0.1, 0.3)	0.3 (0.1, 1.0)
Medium	190 (66.4)	96 (33.4)	1.0 (0.5, 1.8)	0.8 (0.4, 1.9)
Rich	38 (66.7)	19 (33.3)	1.0	1.0

Hosmer-Lemeshow Goodness of fit test = 0.325

*HF* Health facility, *HEW* Health Extension Worker, *COR* crude odds ratio, *AOR* adjusted odds ratio

*p-value ≤0.05 for bivariable analysis

**p-value< 0.05 and ***p-value < 0.001 for multivariable analysis, 1.0 = reference;

## Discussion

Home delivery remains common in Ethiopia but brings many risks for both mothers and their babies. In this study, we characterized the spatial distribution of home deliveries in order to prioritize risks and facilitate geographically based interventions. We found that home delivery after ANC visits shown a spatial variation and remain a public health problem. The occurrence of home deliveries was clustered in rural areas of the Lay Gayint district. Predictors of home deliveries included a number of ANC visits, distance from the nearest health extension worker’s home, being rural, sources of information about the ANC, the decision to choose the place of delivery, knowledge about the place of delivery, and health education.

In this study, 52.7% (95% CI: 48.5, 56.6%) of women delivered at home and without any skilled attendants. The finding is similar to the studies conducted in the Urban Slum of Delhi (India), 53% [16], and Kenya, 53% [17], but is larger than previous studies conducted in Holeta Town, Ethiopia (38.4%) [18], Ghana (21%) [19], Tanzania (25.5%) [20], India (37%) [21], Nigeria (40%) [22], Nepal (45%) [23], and Basra (26.1%) [24]. The possible reasons for home deliveries might be due to the mothers’ perception, sudden onset of labor, distance from a health facility, and inaccessible transportation [5, 20]. However, in this study, the home delivery rate was lower than other studies conducted in Ethiopia, such as in the Sekela district, 87.9% [25], Fogera district, 68.4% [11], and Haromya district, 63.5% [26], as well as studies conducted in rural Tanzania 61% [27], northern Bangladesh 91.3% [28], and rural Nepal 69.2% [29]. These differences might be due to free ANC services in the studied area, and more efficient delivery and ambulance services. Thus, these free services could help to decrease the probability that women will have to give birth at home.

In this study, three significant home delivery clusters were detected within rural areas. This approach explored heterogeneity within residential settings. This finding was inconsistent with a similar study conducted in Indonesia, in which home delivery clusters were rather detected in urban areas [30]. In Ethiopia, the presence of skilled attendants during deliveries is only possible in health facilities, which rural women are therefore less likely to have access too.

This study also identified factors that influence women’s home delivery. Distant women’s home location to the health facility was the main predictor for home delivery. Women from rural areas were four times more likely to choose a home delivery than women from urban areas. This finding was consistent with studies done in Ethiopian districts such as Fogera [11] and Sekela [25], as well as other countries such as Kenya [17], India [21], and Ghana [31]. This may be due to inadequate availability of health services, poor infrastructures, ambulance delays, inaccessibility of information, and low educational status of women from rural areas.

A statistically significant association was found between the distance from a health extension worker’s home and home deliveries, whereby women who lived 2.5–5 km away were two times more likely to deliver at home. This is consistent with another study conducted in Ethiopia [26] and other developing countries such as Kenya [32], Nepal [29], and Zambia [33]. This might be due to inadequate ambulance services and geographic inaccessibility.

Sources of information about ANC services also had a statistically significant association with home deliveries. Namely, women who had received information from health institutions were 68% less likely to deliver at home. This is consistent with another study done in the Haromya district in Ethiopia [26]. A lack of key health information during ANC visits at a health facility could have resulted in women choosing to home deliveries [34].

The number of ANC visits also had a statistically significant association with home deliveries; women who had only one ANC visit were six times more likely to give birth at home. This may be because frequent contact with skilled providers during pregnancy allowed women to acquire more information concerning the importance of the presence of a skilled attendant during childbirth. This is consistent with other studies conducted in other areas of Ethiopia such as Fogera [11], Sekela [25], and Ankasha Guagusa [35], as well as another study conducted in Tanzania [20].

Mothers who did not have any health information exposure were three times more likely to deliver at home. This finding is consistent with studies conducted in Ethiopian districts, such as Fogera and Haromaya [11, 26], northern Bangladesh [28], and Biharamulo, Tanzania [36]. The possible reason for this might be, as pregnant women attend more ANC visits, they have the chance to get health education services. Therefore, those who got this service would give birth at a health facility instead of home.

Home deliveries were 68% less likely when the place of delivery was decided on by both mothers and husbands compared to when the decision was made by mothers only. This finding is consistent with other studies conducted in Addis Ababa, Ethiopia [37], rural Nepal [20], and the Biharamulo district, Tanzania [36]. This is suggestive of the male involvement in the higher rates of health facility deliveries.

These findings detected home delivery clusters that could help to prioritize and develop geographically based interventions to reduce events associated with birth by decreasing home births. This study also identified the clusters of residual values using LISA maps. The significance map also showed the *p*-values on a test of spatial randomness. The p-value could help to show the significance of the Local Moran’s I′. These clusters and spatial outliers’, revealed places of interest that warrant further study. However, the spatial scan statistic might produce large clusters containing many kebele with low home deliveries and distances from women home to health facility were not based on the exact estimate of the actual distance.

## Conclusion

Home deliveries were more common to rural women and remain a public health problem. We found a spatial variation in home deliveries after ANC visits. Our results suggest that improving ANC service utilization and educating mothers and their partners in rural areas may reduce the number of home deliveries and consequently reduce complications associated with it. The increasing availability of Geo-referenced data provided the opportunity to link health facility data with household data, enabling researchers to explore the influences of distance and service quality.

## Data Availability

All relevant data are in the manuscript. However, the minimal data underlying all the findings in the manuscript will be available upon request.

## Abbreviations

ANC: Antenatal care
GIS: Geographic information system
GPS: Global positioning system
HEW: Health extension worker
LLR: LOG Likelihood Ratio
